# Alterations in audiovisual simultaneity perception in amblyopia

**DOI:** 10.1371/journal.pone.0179516

**Published:** 2017-06-09

**Authors:** Michael D. Richards, Herbert C. Goltz, Agnes M. F. Wong

**Affiliations:** 1 Institute of Medical Science, Faculty of Medicine, University of Toronto, Toronto, Canada; 2 Department of Ophthalmology and Vision Sciences, University of Toronto, Toronto, Canada; 3 Department of Ophthalmology and Vision Sciences, The Hospital for Sick Children, Toronto, Canada; 4 Program in Neurosciences and Mental Health, The Hospital for Sick Children, Toronto, Canada; University of Waterloo, CANADA

## Abstract

Amblyopia is a developmental visual impairment that is increasingly recognized to affect higher-level perceptual and multisensory processes. To further investigate the audiovisual (AV) perceptual impairments associated with this condition, we characterized the temporal interval in which asynchronous auditory and visual stimuli are perceived as simultaneous 50% of the time (i.e., the AV simultaneity window). Adults with unilateral amblyopia (n = 17) and visually normal controls (n = 17) judged the simultaneity of a flash and a click presented with both eyes viewing. The signal onset asynchrony (SOA) varied from 0 ms to 450 ms for auditory-lead and visual-lead conditions. A subset of participants with amblyopia (n = 6) was tested monocularly. Compared to the control group, the auditory-lead side of the AV simultaneity window was widened by 48 ms (36%; *p* = 0.002), whereas that of the visual-lead side was widened by 86 ms (37%; *p* = 0.02). The overall mean window width was 500 ms, compared to 366 ms among controls (37% wider; *p* = 0.002). Among participants with amblyopia, the simultaneity window parameters were unchanged by viewing condition, but subgroup analysis revealed differential effects on the parameters by amblyopia severity, etiology, and foveal suppression status. Possible mechanisms to explain these findings include visual temporal uncertainty, interocular perceptual latency asymmetry, and disruption of normal developmental tuning of sensitivity to audiovisual asynchrony.

## Introduction

Amblyopia is a developmental visual impairment caused by abnormal visual experience during a critical period in early childhood. It has a prevalence of 2–4%,[1–7] and is recognized as a leading cause of monocular blindness.[2, 8] Clinically, it presents as a unilateral, or rarely bilateral, reduction in best-corrected visual acuity that cannot be explained solely by a structural eye abnormality. It is often accompanied by one or more factors, most commonly strabismus (eye misalignment) or anisometropia (difference in refractive error between the eyes) that interfere with normal binocular visual experience.[9]

While it is classically understood as a predominantly monocular visual disorder affecting low-level visual functions such as optotype acuity, stereopsis, and contrast sensitivity,[10–13] amblyopia is increasingly recognized to involve deficits in higher-level perceptual processing. Affected individuals show impairments in global shape detection,[14] real-world scene perception,[15] motion processing,[16, 17] and feature counting[18] that affect not only the amblyopic eye, but also often extend to the fellow eye.[19–21] Beyond the purely visual domain, recent work has shown that amblyopia also affects multisensory integration in speech perception, manifest as reduced susceptibility to the McGurk effect, even while viewing with both eyes.[22–24]

Multisensory integration is the process by which information from the various senses is associated and merged into a unified percept. It confers broad advantages in terms of response time[25] and accuracy of discrimination[26] (see Ernst and Bülthoff[27] for review). In infancy, normal visual experience during a critical period is necessary for the emergence of robust integration of auditory and visual signals.[28–30] In turn, audiovisual integration plays an important role in the development of higher level perceptual functions including speech acquisition in infancy[31, 32] and speech comprehension in adulthood.[33–36] Interestingly, deficits in multisensory integration have been increasingly recognized as a feature of various neurodevelopmental disorders, including autism,[37] dyslexia,[38] and schizophrenia,[39, 40] but the mechanism remains elusive.

Visual and auditory stimuli presented in close temporal and spatial correspondence are likely to be perceived as arising from a single event. This process, termed perceptual binding, is a rapid pre-attentive process that occurs without the conscious awareness of the observer, and constitutes a fundamental rule for learning associations between stimuli.[36, 41, 42] Neuroimaging studies indicate that the temporal correspondence of auditory and visual speech stimuli activates a broad network, including the superior colliculus (SC), anterior insula, and anterior intraparietal sulcus (IPS), while perceptual fusion (e.g. as in the McGurk effect) is associated with activation in the multisensory superior temporal sulcus (mSTS), the middle IPS, and regions of the primary auditory cortex.[43–45] Similar studies of non-speech stimuli (e.g. click-flash pairs) have shown that temporal correspondence of simple AV stimuli activates the SC, mSTS, IPS, and insula,[46] while detection or perception of asynchrony is associated with activation of an extensive network including the insula, posterior parietal, and prefrontal regions, with the right insula being involved most significantly.[47] Furthermore, Noesselt at al.[48] showed that temporal correspondence of simple AV stimuli not only activates the mSTS, but also affects activity in the primary auditory and visual cortices, likely by a feedback mechanism from the mSTS.

The temporal interval during which separate visual and auditory stimuli are perceived reliably as simultaneous is termed the audiovisual (AV) simultaneity window, and reflects an equilibrium between the sensitivity to signal asynchrony (which narrows the AV simultaneity window) and the tendency toward perceptual binding (which widens the AV simultaneity window). It is measured using a single-interval forced-choice simultaneity judgment task for AV stimulus pairs presented with varying signal onset asynchrony (SOA). It typically has a bell-shaped distribution with a slight skew toward the visual-lead side of objective simultaneity.[49–51] Furthermore, AV stimuli are typically perceived as maximally simultaneous when the visual stimulus slightly precedes the sound. This visual-lead shift in the point of subjective simultaneity (PSS) is commonly believed to reflect either tuning to the natural condition in which light waves reach the eyes before sound waves reach the ears, or the neural delay related to slower processing of visual signals.[52] The AV simultaneity window progressively narrows on both auditory-lead and visual-lead sides from childhood through adolescence, reaching the adult shape by 9 to 17 years of age.[53–56] Interestingly, individuals with a narrower AV simultaneity window, particularly on the visual-lead side, experience a stronger McGurk effect, suggesting that the AV simultaneity window may be an index of broader audiovisual integrative function.[57]

For an individual with a developmentally normal sensorium, the overall width of the AV simultaneity window is not fixed, but varies depending on the characteristics of the stimuli. Complex stimuli such as natural speech and audiovisual stimuli with high semantic congruency result in a wider AV simultaneity window than simple flash-beep stimuli.[50, 58] Increased spatial separation between the paired stimuli,[51, 59] as well as availability of visual predictive information about when to expect an audiovisual event to occur,[60] result in a narrower AV simultaneity window. Its width can be further narrowed by various forms of perceptual learning—short-term audiovisual and visual-only training with feedback,[61, 62] long-term musical training,[63] and video gaming experience.[64] In addition to the width of the AV simultaneity window, its peak, or point of subjective simultaneity is also variable. Repeated exposure to asynchronous stimuli shifts it toward the trained asynchrony in a process termed temporal recalibration.[65–67] Furthermore, the presence of an additional visual stimulus that closely precedes or follows a synchronous AV pair biases the PSS away from the additional stimulus.[68]

Abnormal early visual experience has been shown to affect multisensory processing. Adults with early pattern vision deprivation from bilateral congenital cataracts have an AV simultaneity window that is selectively broadened on the visual-lead side,[69] as well as diminished audiovisual interaction in speech perception,[30] and a shift in attentional balance toward audition.[70] In contrast, the AV simultaneity window of adults with unilateral congenital cataract is symmetrically broadened, similar to that seen in typically-developing children.[69] Audiovisual interactions have also been studied in monocular adults with a history of early enucleation. Like those with unilateral amblyopia, this population shows reduced susceptibility to the McGurk effect, but demonstrate normal responses to illusions involving temporal audiovisual integration such as the sound-induced flash illusion and AV simultaneity judgments.[71] This suggests that AV integration deficits may be specific to the nature of the visual sensory disturbance during the critical period.

Despite its relatively high prevalence, much less is known about the extent of the multisensory deficits in unilateral amblyopia from strabismus and anisometropia. Specifically, it is unclear whether the audiovisual integration deficits in these forms of amblyopia are specific to speech, or whether they reflect a broader impairment in multisensory processing. Evidence from visually normal adults suggests that susceptibility to the McGurk effect is correlated with other indices of temporal audiovisual integration.[57] One such index is the AV simultaneity window. Visually normal individuals with lower susceptibility to the McGurk effect have a wider AV simultaneity window, indicating altered processing of asynchronous multimodal signals.[57] Based on this evidence from visually normal adults and our previous studies showing that adults with amblyopia are less susceptible to the McGurk effect,[23, 24] we hypothesized that unilateral amblyopia will also show a symmetrically broadened AV simultaneity window under binocular and monocular viewing conditions, indicating a higher-level alteration in audiovisual integration that is generalized beyond speech.

## Materials and methods

### Participants

Participants were adults aged 18 to 48 years, with no history of neurological, auditory, or visual disorders other than amblyopia, strabismus, or ametropia. Each participant was assessed by a certified orthoptist or ophthalmologist to document visual acuity (standard ETDRS chart), stereoacuity (Randot circles test and Titmus fly test), binocularity (Worth 4-dot test), eye alignment (cover-uncover and alternate cover tests), and refractive correction. Amblyopia was defined as a visual acuity of 0.18 logMAR (20/40) or worse in the amblyopic eye, and an inter-ocular difference of at least 0.2 logMAR (2 lines on the ETDRS chart). Anisometropic amblyopia was defined as an inter-ocular difference of 1 diopter (D) or more in either spherical equivalent or astigmatic correction. Strabismic amblyopia was defined as any manifest deviation on cover testing in the absence of anisometropia. Mixed amblyopia was defined as the presence of both anisometropia and a manifest deviation of 8 prism diopters or more. Visually normal was defined as visual acuity of at least 0.1 logMAR (20/25) in each eye. All participants completed a hearing test on a commercially-available screening audiometer (model MA 27, MAICO Diagnostics, Eden Prairie, MN, USA) with circumaural headphones (model TDH 39, MAICO Diagnostics, Eden Prairie, MN, USA) to ensure reliable responses to low level (≤25 dB) pure tones at a standard set of frequencies (0.5, 1, 2, and 4 kHz).[72] Participants were excluded if they had a history of any other ocular pathology, previous intraocular surgery, high ametropia (hyperopia > +5D or myopia > -6D), hearing impairment, neurological disease, or neurodevelopmental disorder. Written informed consent was obtained from all participants. The study was approved by the Research Ethics Board at The Hospital for Sick Children, and all protocols adhered to the tenets of the Declaration of Helsinki.

Participants were recruited from November 2014 to February 2016 through flyers posted on hospital property and advertisements posted on the social media websites Craigslist.ca and Kijiji.ca. Of 26 individuals with amblyopia recruited, 17 passed the screening examinations and participated in the study (3 males, mean age: 29 years, range: 19–48 years). An equal number of visually normal naive control participants were recruited in a similar fashion (4 males, mean age: 29 years, range: 22–47 years). The clinical characteristics of the participants with amblyopia are summarized in Table 1.

**Table 1:** Characteristics of participants with amblyopia.

			Visual acuity (logMAR)		Refractive correction			
Participant	Age	Subtype	RE	LE	RE	LE	Stereopsis (arcsec)	Worth 4-dot response
1	29	Strab	0.00	1.00	None	None	Not measurable	LE suppressed
2	22	Aniso	0.00	0.48	-1.50 +0.50 x 80	+1.00 +1.25 x 95	200	Fused
3	48	Aniso	0.70	0.00	+2.25 +0.25 x 174	-0.75	3000	Fused
4	36	Aniso	0.00	0.40	-1.00	+1.00	140	Fused
5	29	Aniso	0.48	-0.10	-5.00	-1.25	3000	Fused
6	23	Aniso	-0.10	0.48	-2.25	+0.25 +2.25 x 85	200	Fused
7	29	Aniso	0.10	0.70	-1.50 +1.50 x 100	-3.00 +1.50 x 93	Not measurable	LE suppressed
8	32	Strab	-0.10	0.18	None	None	70	Fused
9	29	Mixed	0.00	1.00	Plano	+3.50 +2.00 x 90	Not measurable	LE suppressed
10	19	Aniso	0.00	0.18	-0.75 +2.00 x 84	-2.75 +4.50 x 99	40	Fused
11	37	Mixed	-0.10	1.30	-1.00	+6.00 +2.50 x 120	Not measurable	LE suppressed
12	32	Aniso	-0.10	0.54	Plano	+2.00 +2.00 x 124	140	Fused
13	23	Strab	0.20	0.00	+0.50 +0.50 x 28	+1.25 +0.50 x 88	Not measurable	Diplopic
14	44	Mixed	0.90	0.00	+6.00+1.25x75	-0.75	Not measurable	RE suppressed
15	22	Aniso	1.1	-0.10	-6.00+0.75x174	-4.50+0.50x75	3000	Fused
16	19	Mixed	0.48	0.00	+3.00+1.00x130	+4.25	3000	Fused
17	27	Strab	0.00	0.48	-6.25 +1.00 x 45	-5.50 +1.25 x 135	200	Fused

Abbreviations: RE, right eye; LE, left eye; Aniso, anisometropia; Strab, strabismic.

### Apparatus and stimuli

Experiments were performed in a dark, sound attenuating chamber (internal dimensions 2.0 x 2.1 x 2.2 m) lined with 5 cm acoustic wedge foam (Foam Factory, Macomb, MI, USA). The background noise was 39.0 dBA sound pressure level (SPL). Visual stimuli were gray Gaussian blobs (6 SD = 4°) presented centrally for 33 ms (2 frames at 60 Hz) on a 165 cm LED monitor (NEC, model E654, Tokyo, Japan). Auditory stimuli were 32 ms white noise click trains (including a 2 ms sigmoid on/off ramp) presented at 62.0 dBA SPL via stereo speakers (HP Inc., model BR387AA#ABA, Palo Alto, CA, USA) mounted on either side of the monitor. Stimuli were created digitally and controlled using a custom-written program, and participant responses were collected directly via a gamepad (Logitech, model F710, Newark, CA, USA). The visual and acoustic signals were horizontally aligned at eye level of the seated participant, and relative timing was confirmed with an oscilloscope.

### Procedure

**Fig 1:**
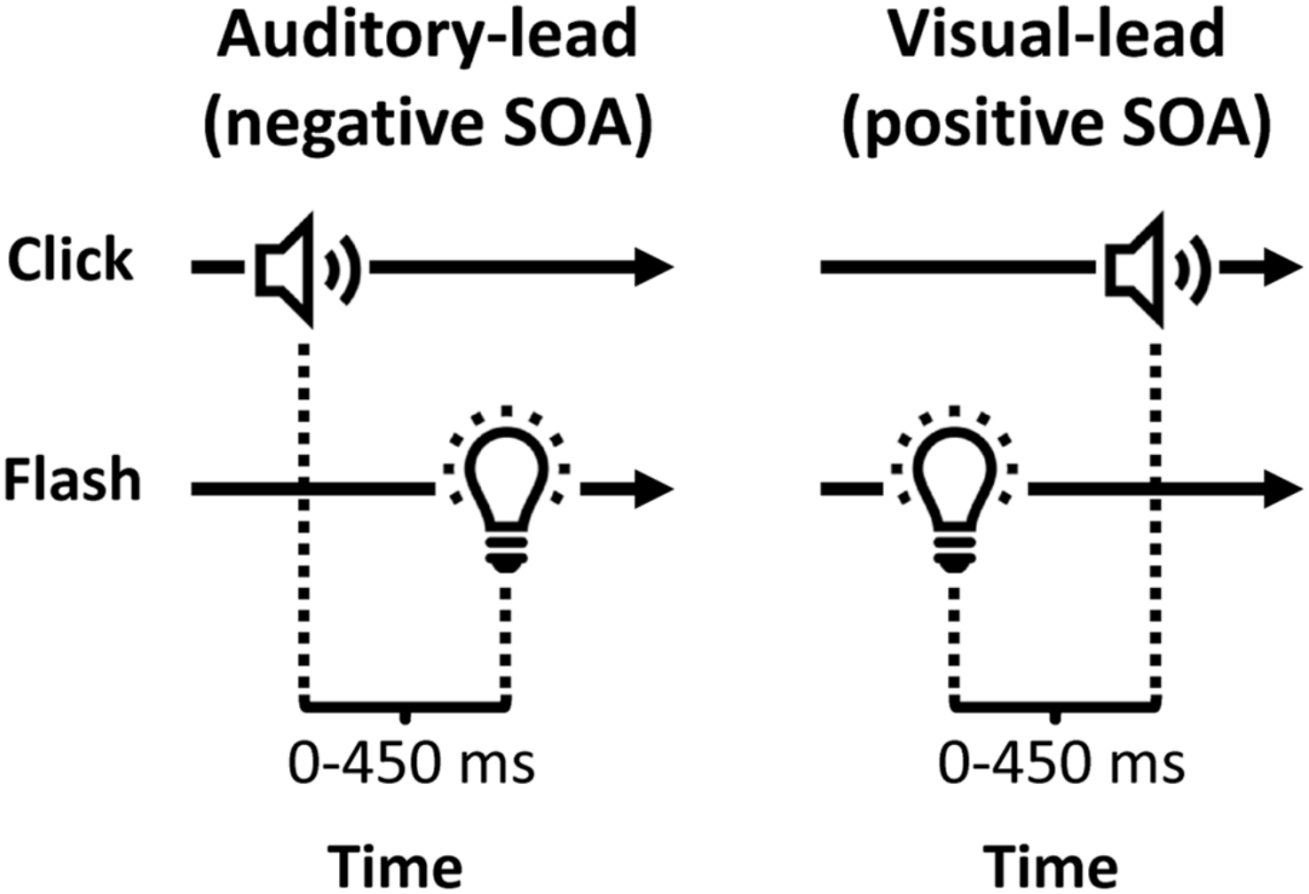
Schematic diagram of signal onset asynchronies (SOA) for auditory-lead and visual-lead conditions.

The AV simultaneity window was characterized using a two-alternative forced-choice (2AFC) simultaneity judgement task. With the head stabilized on a chinrest 65 cm from the LED monitor, participants were required to fixate a central red dot on the monitor (0.7°) and press a button on the gamepad to initiate each trial. Following a random interval of 500 to 1500 ms during which the screen was dark, a flash-click pair was presented, and the participant indicated whether the two stimuli were “simultaneous” or “not simultaneous”. The signal onset asynchrony (SOA) was varied from -450 ms (auditory stimulus presented first, i.e., auditory-lead) to +450 ms (visual stimulus presented first, i.e., visual-lead) in 75 ms increments (i.e. -450, -375, -300, -225, -150, -75, 0, +75, +150, +225, +300, +375, +450 ms) for a total of 13 SOA levels (Fig 1). There were 20 trials for each SOA level, randomly interleaved in a single block, typically taking 12–15 minutes to complete. Data were collected under binocular viewing conditions for all participants. Data were also collected under amblyopic eye and fellow eye monocular viewing conditions for a subset of 6 participants with amblyopia to determine if any group effects were dependent on viewing condition.

### Analysis

**Fig 2:**
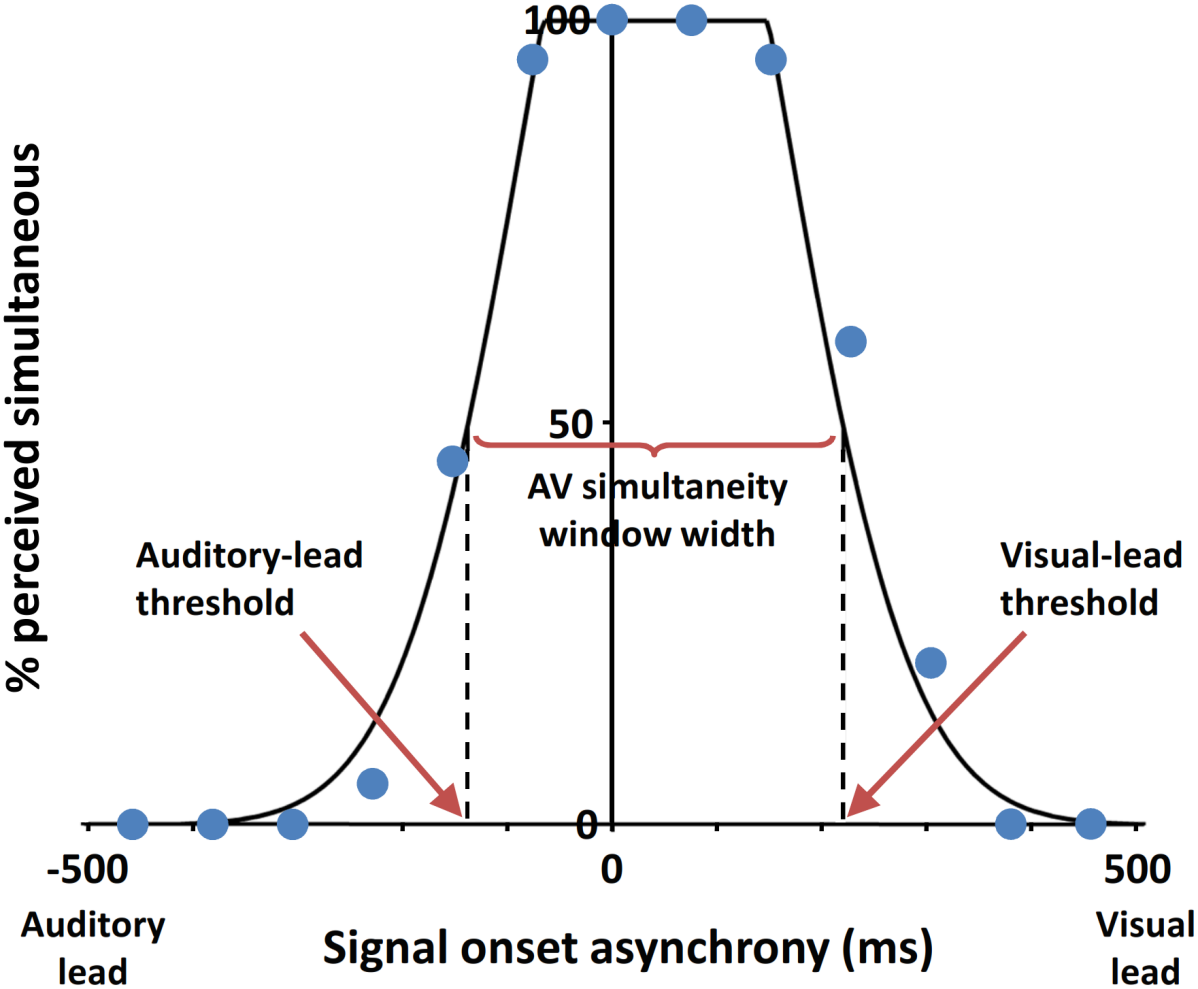
Sample audiovisual simultaneity judgment data from a visually normal control participant, fitted with a truncated Gaussian function by the maximum likelihood method. The psychometric parameters (i.e., AV simultaneity window width, auditory-lead threshold and visual-lead threshold), were estimated at the 50% simultaneous response level.

The proportion of “simultaneous” responses was calculated for each SOA, and the response distribution was fitted with a previously described truncated Gaussian function using the maximum likelihood method.[66] The correlation coefficient of the fit was ≥0.93 for each individual. The *AV simultaneity window width* was defined as the width of the fitted function at the 50% simultaneous response level, with the SOA to the left and right of 0 ms (i.e. physical simultaneity) representing the *auditory-lead threshold* and *visual-lead threshold*, respectively. The *point of subjective simultaneity (PSS)* was defined as the mean of the fitted truncated Gaussian function. Group parameters were calculated as the arithmetic means of the individual participant parameters. Sample data with fitted function are shown in Fig 2. All curve fitting and parameter calculations were done using MATLAB version 2011b (Mathworks, Inc., Natick, MA, USA).

Performance parameters (i.e., auditory-lead threshold, visual-lead threshold, AV simultaneity window width, and PSS) were compared between groups using one-way analysis of variance (ANOVA) and Tukey post-hoc multiple comparisons. Homogeneity of variances was verified in each case by Levene’s test. Subgroup analyses were performed based on 4 common clinical factors in amblyopia: 1) severity of the monocular acuity deficit, 2) presumed etiology, 3) presence or absence of foveal suppression, and 4) level of stereopsis. Amblyopia severity was classified as moderate if the acuity was ≤ 0.6 logMAR in the amblyopic eye, and as severe if the acuity was >0.6 logMAR.[9] Presumed etiology was classified as either anisometropic or strabismic/mixed. Foveal suppression status was classified as suppressed or non-suppressed based on results from the Worth 4-dot test. Level of stereopsis was classified as fine (i.e., some Randot circles; ≤400 seconds of arc) or poor (i.e., no Randot circles). Associations between the 4 clinical factors were assessed using 2x2 contingency tables and the phi coefficient (*Φ*). All statistics were computed using IBM SPSS Statistics version 22 (Armonk, NY, USA). Statistical significance was defined as *p* < 0.05.

## Results

### Binocular viewing condition

#### Main group analysis

**Fig 3:**
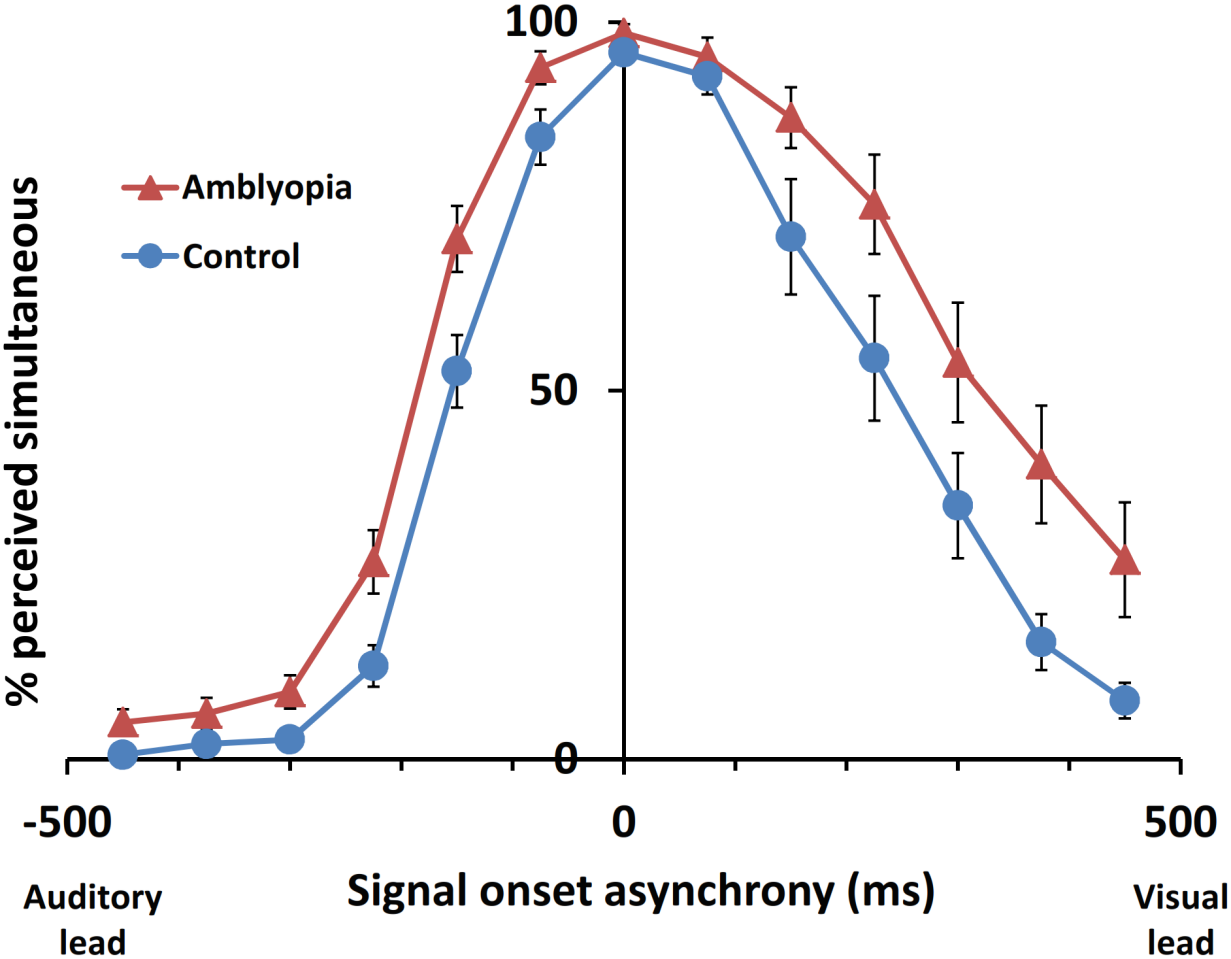
Main group analysis for audiovisual simultaneity judgment responses with both eyes viewing as a function of SOA. Comparison between control (n = 17) and amblyopia (n = 17) participant groups. Error bars represent standard error of the mean.

The AV simultaneity window in adults with unilateral amblyopia was broadened by 134 ms, or 37%, compared to control participants (*F*_(1,32)_ = 11.313, *p* = 0.002) when viewing binocularly (Fig 3 and Table 2). The auditory-lead side of the AV simultaneity window was wider by 48 ms (36%; *F*_(1,32)_ = 11.012, *p* = 0.002), and the visual-lead side was wider by 86 ms (37%; *F*_(1,32)_ = 6.00, *p* = 0.02). There was no significant difference in the PSS between the control and amblyopia group.

**Table 2:** AV simultaneity window parameters by main group.

	SOA, mean ± SD (ms)			
Performance parameter	Control (n = 17)	Amblyopia (n = 17)	*F*_(1,32)_	*p*-value
**Auditory-lead threshold**	-136 ± 34	-183 ± 49*	11.012	0.002
**Visual-lead threshold**	231 ± 83	317 ± 119*	6.000	0.020
**AV simultaneity window width**	366 ± 91	500 ± 136*	11.313	0.002
**PSS**	47 ± 44	67 ± 60	1.131	0.295

Abbreviations:

* *p* < 0.05 (one-way ANOVA); SOA, signal onset asynchrony; SD, standard deviation.

### Subgroup analysis by clinical factors

#### 1. Amblyopia severity

**Fig 4:**
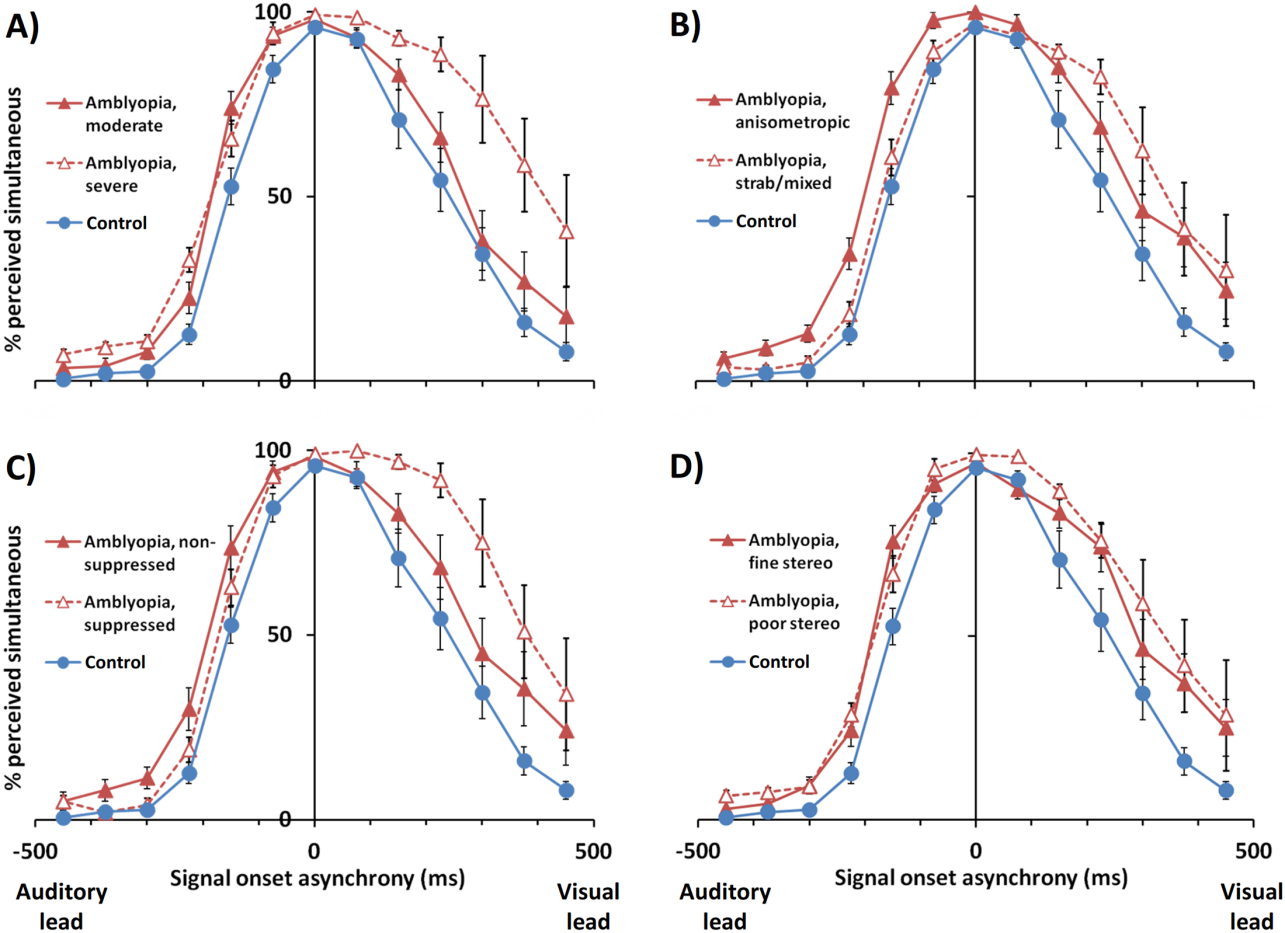
Subgroup analyses for audiovisual simultaneity judgment responses with both eyes viewing as a function of SOA. (A) Comparison by amblyopia severity. (B) Comparison by presumed etiology. (C) Comparison by foveal suppression status. (D) Comparison by level of stereopsis. Error bars represent standard error of the mean.

Results of the subgroup analysis by amblyopia severity are summarized in Table 3 and Fig 4A. In the moderate amblyopia subgroup (n = 10), the auditory-lead threshold was broadened by 45 ms (33%; *p* = 0.032), but the other parameters (visual-lead threshold, AV simultaneity window, and PSS) were not significantly different from the control group. In the severe amblyopia subgroup (n = 7), three parameters were broadened compared to the control group: the auditory-lead threshold by 51 ms (38%; *p* = 0.030), the visual-lead threshold by 155 ms (67%; *p* = 0.003), and the AV simultaneity window by 207 ms (57%; *p* = 0.001). The PSS in the severe amblyopia group showed a non-significant trend toward a visual-lead shift compared to the control group (*p* = 0.064).

**Table 3:** AV simultaneity window parameters by amblyopia severity.

	SOA, mean ± SD (ms)				
Performance parameter	Control (n = 17)	Amblyopia		*F*_(2,31)_	Omnibus *p*-value
		Moderate (n = 10)	Severe (n = 7)		
**Auditory-lead threshold**	-136 ± 34	-180 ± 39*	-186 ± 63*	5.393	0.010
**Visual-lead threshold**	231 ± 83	268 ± 103	386 ± 111*†	6.700	0.004
**AV simultaneity window width**	366 ± 91	448 ± 126	572 ± 122*	9.120	0.001
**PSS**	47 ± 44	44 ± 45	100 ± 67	3.281	0.051

Abbreviations:

* *p* < 0.05 (vs. Control group *post hoc*);

^†^ *p* < 0.05 (vs. Moderate group *post hoc*); SOA, signal onset asynchrony; SD, standard deviation.

Within the amblyopia group (i.e., moderate vs. severe), severity was significantly related to only the visual-lead threshold, with those classified as severe having a threshold 118 ms wider compared those classified as moderate (*p* = 0.043). Severe amblyopia also showed non-significant trends toward a wider simultaneity window (*p* = 0.068) and a visual-lead shifted PSS (*p* = 0.071) compared to the moderate group.

#### 2. Amblyopia etiology

Results of the subgroup analysis by amblyopia etiology are summarized in Table 4 and Fig 4B. In the anisometropic subgroup, the auditory-lead threshold was broadened by 75 ms (56%; *p* < 0.001) and the AV simultaneity window was broadened by 134 ms (37%; *p* = 0.025), but the visual-lead threshold and PSS were not significantly different from the control group. In the strabismic/mixed subgroup, the visual-lead threshold was broadened by 116 ms (32%, *p* = 0.032), and the AV simultaneity window was broadened by 133 ms (36%; *p* = 0.033), but unlike the anisometropic group, the auditory-lead threshold was not significantly different compared to the control group. There was a non-significant trend toward a visual-lead shifted PSS in the strabismic/mixed group compared to the control group (*p* = 0.064).

**Table 4:** AV simultaneity window parameters by amblyopia etiology.

	SOA, mean ± SD (ms)				
Performance parameter	Control (n = 17)	Amblyopia		*F*_(2,31)_	Omnibus *p*-value
		Aniso (n = 9)	Strab/mixed (n = 8)		
**Auditory-lead threshold**	-136 ± 34	-210 ± 44*†	-153 ± 34	12.165	<0.001
**Visual-lead threshold**	231 ± 83	289 ± 107	348 ± 131*	3.689	0.037
**AV simultaneity window width**	366 ± 91	500 ± 134*	500 ± 147*	5.480	0.009
**PSS**	47 ± 44	40 ± 47	97 ± 61	3.513	0.042

Abbreviations:

* *p* < 0.05 (vs. Control group *post hoc*);

^†^ *p* < 0.05 (vs. Strab/mixed group *post hoc*); SOA, signal onset asynchrony; SD, standard deviation; Aniso, anisometropic; Strab, strabismic.

Within the amblyopia group (i.e. anisometropic vs. strabismic/mixed), etiology was significantly related to the auditory-lead threshold, with those classified as anisometropic having an threshold 57 ms wider compared those classified as strabismic/mixed (*p* = 0.009). The PSS in the strabismic/mixed group also showed a non-significant trend toward a visual-lead shift compared to the anisometropic group (*p* = 0.058).

#### 3. Foveal suppression status

Results of the subgroup analysis by foveal suppression status are summarized in Table 5 and Fig 4C. In the non-suppressed subgroup, the auditory-lead threshold was broadened by 20 ms (43%; *p* = 0.002) and the AV simultaneity window was broadened by 116 ms (32%; *p* = 0.033), but the visual-lead threshold and PSS were not significantly different from the control group. In the suppressed subgroup, the visual-lead threshold was broadened by 156 ms (68%, *p* = 0.011), the AV simultaneity window was widened by 177 ms (48%; *p* = 0.014), and the PSS was shifted toward by visual-lead condition by 68 ms (*p* = 0.025), but the auditory-lead threshold was not significantly different compared to the control group.

**Table 5:** AV simultaneity window parameters by suppression status.

	SOA, mean ± SD (ms)				
Performance parameter	Control (n = 17)	Amblyopia		*F*_(2,31)_	Omnibus *p*-value
		Non-suppressed (n = 12)	Suppressed (n = 5)		
**Auditory-lead threshold**	-136 ± 34	-195 ± 53*	-156 ± 20	7.432	0.002
**Visual-lead threshold**	231 ± 82	287 ± 112	387 ± 114*	5.041	0.013
**AV simultaneity window width**	366 ± 91	481 ± 149*	543 ± 99*	6.146	0.006
**PSS**	47 ± 44	46 ± 47	115 ± 65*†	4.286	0.023

Abbreviations:

* *p* < 0.05 (vs. Control group *post hoc*);

^†^ *p* < 0.05 (vs. Non-suppressed group *post hoc*); SOA, signal onset asynchrony; SD, standard deviation; W4D, Worth 4-dot test

Within the amblyopia group, suppression status was significantly related to the PSS only, with those classified as suppressed having a PSS shifted 69 ms toward the visual-lead condition compared those classified as non-suppressed (*p* = 0.030).

#### 4. Stereopsis level

Results of the subgroup analysis by stereopsis level are summarized in Table 6 and Fig 4D. In the subgroup with fine stereopsis, none of the simultaneity window parameters were significantly different from the control group, although there was a trend toward broadening of the auditory-lead threshold that did not reach significance in post-hoc testing (*p* = 0.055). In the subgroup with gross stereopsis, the auditory-lead threshold was broadened by 49 ms (36%, *p* = 0.019), the visual-lead threshold was broadened by 103 ms (45%, *p* = 0.045), and the AV simultaneity window was broadened by 151 ms (41%; *p* = 0.007), but the PSS was not shifted compared to the control group.

**Table 6:** AV simultaneity window parameters by stereopsis level.

	SOA, mean ± SD (ms)				
Performance parameter	Control (n = 17)	Amblyopia		*F*_(2,31)_	Omnibus *p*-value
		Fine stereopsis (n = 7)	Poor stereopsis (n = 10)		
**Auditory-lead threshold**	-136 ± 34	-182 ± 42	-184 ± 55*	5.343	0.010
**Visual-lead threshold**	231 ± 83	293 ± 112	333 ± 126*	3.289	0.051
**AV simultaneity window width**	366 ± 91	475 ± 143	518 ± 136*	5.861	0.007
**PSS**	47 ± 44	55 ± 45	75 ± 70	0.828	0.447

Abbreviations:

* *p* < 0.05 (vs. Control group *post hoc*); SOA, signal onset asynchrony; SD, standard deviation

Within the amblyopia group, level of stereopsis was not significantly related to any simultaneity window parameters.

#### 5. Associations between clinical factors

Participants with strabismic/mixed amblyopia were significantly more likely to exhibit foveal suppression on the Worth 4-dot test compared to those with anisometropic amblyopia (*Φ* = 0.685, *p* = 0.005). Etiology was not significantly associated with amblyopia severity (*Φ* = 0.169, *p* = 0.486) or stereopsis level (*Φ* = 0.310, *p* = 0.201). Participants with severe amblyopia were significantly more likely to demonstrate foveal suppression (*Φ* = 0.509, *p* = 0.036) and to have poor stereopsis (*Φ* = 0.700, *p* = 0.004) compared to those with moderate amblyopia. Participants with foveal suppression on the Worth 4-dot test were significantly more likely to have poor stereopsis compared to those with a non-suppressed response (*Φ* = 0.540, *p* = 0.026).

### Monocular viewing conditions

**Fig 5:**
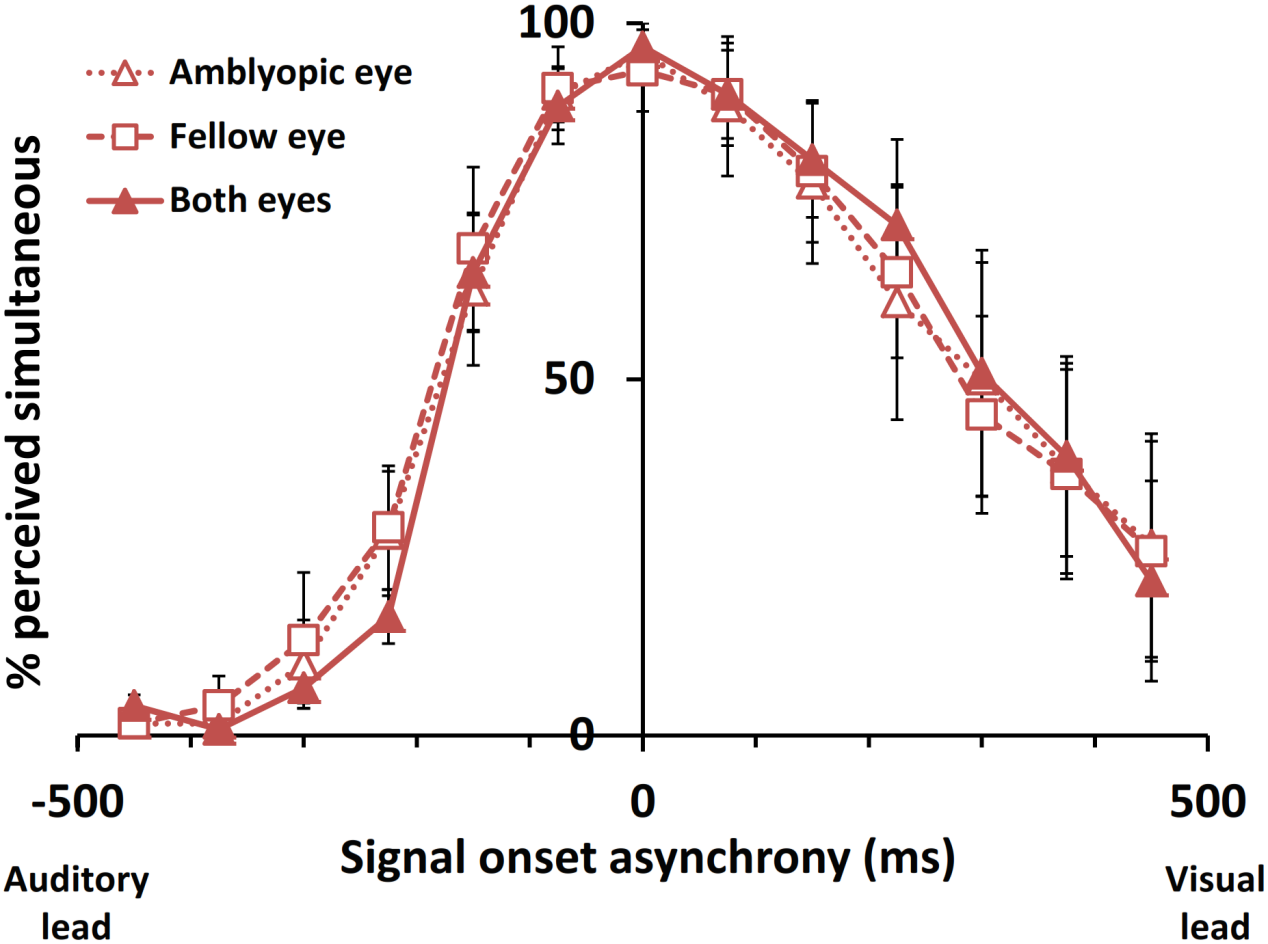
The audiovisual simultaneity window for binocular and monocular viewing conditions among participants with amblyopia. There were no significant differences between viewing conditions (n = 6). Error bars represent standard error of the mean.

A subset of 6 participants with amblyopia was tested under monocular amblyopic eye-only and fellow eye-only viewing conditions. The mean “simultaneous” response percentages are plotted by SOA in Fig 5. Repeated measures ANOVAs, summarized in Table 7, showed no significant differences in any performance parameters across viewing conditions among participants with amblyopia.

**Table 7:** Comparison of AV simultaneity window parameters by viewing condition for participants with amblyopia (Repeated measures ANOVA).

	SOA, mean ± SD (ms)				
Performance parameter	Both eyes	Amblyopic eye	Fellow eye	*F*_(2,10)_	Omnibus *p*-value
**Auditory-lead threshold**	-158 ± 40	-166 ± 53	-177 ± 73	0.331	0.726
**Visual-lead threshold**	304 ± 155	283 ± 145	279 ± 119	0.607	0.564
**AV simultaneity window width**	462 ± 157	449 ± 177	456 ± 144	0.054	0.948
**PSS**	73 ± 82	58 ± 64	51 ± 67	1.244	0.329

Abbreviations: SOA, signal onset asynchrony; SD, standard deviation

## Discussion

We characterized the AV simultaneity window in adults with unilateral amblyopia and in visually normal control participants using a simultaneity judgment task. The window parameter values obtained among control participants were very similar to those previously published for similar experimental protocols.[50, 57, 73, 74] With both eyes viewing, the window was wider in participants with amblyopia on both the auditory-lead and visual-lead sides. The broadening of the simultaneity window among participants with amblyopia was similar among amblyopic eye only, fellow eye only, and binocular viewing conditions, suggesting that these perceptual differences may involve an abnormal central multisensory network for temporal processing. The results are similar to those reported for adults with early monocular deprivation from congenital cataract,[69] and demonstrate that the abnormalities in audiovisual integration in the most prevalent forms of amblyopia are not specific to the McGurk effect (i.e., AV speech perception)[22–24], but generalize to simultaneity judgements of simple, non-speech stimuli.

Subgroup analyses of the participants with amblyopia by their clinical characteristics showed several differentiating patterns. The auditory-lead side of the simultaneity window varied with etiology, with significant broadening seen in the anisometropic group. In contrast, the visual-lead side varied with severity, with significant broadening seen in the severe group. The PSS is a composite of the auditory-lead and visual-lead threshold values, and as such, exhibited an intermediate response: the PSS trended toward visual-lead shifts in the strabismic/mixed group and in the severe group, and showed a significant visual-lead shift in the foveal suppression group.

A major distinction between strabmismic and anisometropic amblyopia is the difference in binocular function.[75] Strabismic and mixed mechanism amblyopia tend to show stronger suppression and poorer stereopsis than anisometropic amblyopia.[75–77] Interestingly, the clinical characteristics associated with a broadened visual-lead threshold and visual-lead shifted PSS in this study are also those known to indicate poor binocularity: strabismic/mixed etiology, foveal suppression, and a severe monocular acuity deficit. Conversely, anisometropic etiology is known to indicate relatively better binocular function, and was the only clinical characteristic positively associated with a broadened auditory-lead threshold in this study.

While anisometropic and strabismic/mixed etiologies were distinguished by their effect on the auditory-lead side of the AV simultaneity window, several observations are noteworthy (see Table 4 and Fig 4b). First, the width of the AV simultaneity window among the two etiology groups was the same. Second, the magnitude and direction of the differences in the auditory-lead threshold, visual-lead threshold, and PSS (i.e. the midpoint of the two thresholds) between the two etiology groups were nearly identical (i.e., 57–58 ms toward the visual-lead side), suggesting a shift in the function rather than a widening of the visual-lead side. Third, these effects are unlikely to be confounded by amblyopia severity, as there was no statistical association between etiology and severity in the study sample. Taken together, these observations suggest that two distinct mechanisms may be at play: that amblyopia in the absence of significant strabismus or suppression (e.g., anisometropic amblyopia) leads to a symmetric broadening of the AV simultaneity window without shifting the PSS, and that it is the overlay of significant strabismus or suppression (e.g. strabismic/mixed amblyopia) that shifts the PSS toward the visual-lead condition. A symmetric broadening of the AV simultaneity window without a shift in PSS has also been observed in unilateral deprivational amblyopia.[69] Importantly, deprivational and anisometropic amblyopia share image degradation as common factor, and exhibit similarities on psychophysical tests of spatial acuity and binocularity,[75] lending further support to the hypothesis outlined above. Because of the statistical associations between the clinical characteristics in the study sample, the results must be interpreted with caution. Amblyopia severity was significantly associated with every clinical characteristic except etiology, meaning that interpretation of the subgroup analyses for suppression and stereopsis is confounded by unbalanced severity between groups. Some variables may also reflect clinical factors, such as age of onset, which cannot generally be determined accurately. Strabismus, for example, accounts for the majority of amblyopia cases under age 3 years, while anisometropia becomes an etiologic factor primarily after age 3.[78] It is also likely that amblyopic etiology, suppression, stereopsis, and severity constitute overlapping measures of common factors such as binocular function, or age of onset, although their relations and these interactions are undoubtedly complex.[75]

In visually normal individuals, the width of the AV simultaneity window and PSS are not only determined by sensory physiology, but are also modulated by cognitive factors such as attention, and a decisional bias toward simultaneity.[74] Attending to either vision or audition has been shown to shift the PSS away from the attended modality in a phenomenon termed *prior entry*.[79]. While it is possible that amblyopia is associated with an attentional shift toward audition[70], others have determined that the magnitude of the prior entry effect in this task among visually normal individuals is only 14 ms–far less than the 69 ms shift observed in the foveal suppression group in this study.[74] Decisional bias toward simultaneity (i.e. shift in criterion for the unity assumption) would have the effect of widening both the auditory-lead and visual-lead sides of the window without shifting the PSS.[80] However, it has been shown that within individuals, the width of the simultaneity window is stable over time[73] and unaffected by the range of SOAs tested, suggesting that this parameter reflects perceptual rather than decisional factors.[51, 53] Indeed, if a decisional bias toward unity was the cause of a widened simultaneity window in amblyopia, one might also expect that susceptibility to the McGurk effect would also be heightened, but this is not the case.[22, 23]

Multiple non-cognitive factors may also contribute to the main and subgroup differences in audiovisual temporal perception described in this study. Hypothetically, widening of the simultaneity window could result from strengthened multisensory perceptual binding. As with decisional bias toward unity, however, the heightened McGurk effect expected from enhanced AV perceptual binding has not been observed in amblyopia.[22–24] Rather, the accompaniment of a wide simultaneity window in amblyopia with low susceptibility to the McGurk is akin to the relation observed in visually normal individuals,[57] and suggests an impairment in the ability to resolve asynchronous AV pairs as unique events. A possible mechanism for such an impairment is temporal uncertainty in the visual domain. Assuming that decisional and criterion factors are unchanged, less precise visual temporal information would reduce the precision of AV asynchrony detection, and widen the simultaneity window. Indeed, evidence for temporal uncertainty in amblyopia exists. Spang and Fahle[81] reported reduced visual temporal resolution in the amblyopic eyes of anisometropic and strabismic participants, and that the temporal deficit correlated with amblyopia severity as in the present study. Huang and others[82] employed a synchrony detection task to demonstrate a foveal temporal processing impairment in the amblyopic eye of strabismic and anisometropic participants. Impaired temporal processing is also evident in the fellow eye in strabismic amblyopia when the judgment of temporal order requires interhemispheric transmission across the corpus callosum.[83] Visual temporal uncertainty such as that demonstrated in amblyopia can be expected to have downstream effects on multisensory processes, including AV asynchrony detection, dependent on visual input.

As discussed above, the PSS shift toward visual-lead SOAs among participants with foveal suppression was larger than that which is solely attributable to attentional effects.[74] PSS shifts of more comparable magnitude, however, have been observed in normal adults as a result of temporal recalibration to constant asynchrony.[66] This phenomenon is likely an important mechanism to deal with the natural physical and neural asynchrony in auditory and visual signals, and presents a possible mechanism for the PSS shifts observed in amblyopia. In visually normal adults, the first peak cortical evoked response occurs 75 ms after onset of an auditory stimulus and 104 ms after onset of a visual stimulus, resulting in a neural asynchrony of about 30 ms even under ideal conditions.[84] In amblyopia, however, cortical response latencies from the affected eye are increased compared to the fellow eye.[85, 86] This transmission latency difference may be another source of temporal uncertainty and act as the perceptual stimulus to shift the PSS toward visual-lead SOAs. Indeed, evidence for a significant interocular perceptual latency difference in amblyopia is provided by the observation of a spontaneous Pulfrich effect in some observers with amblyopia.[87] Another possible explanation for the PSS shift in amblyopia is that suppression and poor stereopsis may interfere with the normal ability to account for sound velocity and source distance when making AV simultaneity judgments.[88, 89] This explanation, however, is unlikely, as monocular adults who lost one eye at an early age perform as normal controls in this task.[71]

If the putative audiovisual temporal correspondence detector were intact in amblyopia, one could reasonably speculate that occlusion of the affected eye would eliminate the temporal uncertainty and perceptual latency, and normalize the AV simultaneity window parameters. However, we found viewing condition had no significant effect on the simultaneity window parameters. This result agrees with the findings in deprivational amblyopia,[69] and suggests that the abnormality in audiovisual simultaneity judgment is not solely a result of amblyopic visual input, but that it involves a central alteration in the capacity to process audiovisual temporal information. Furthermore, this interpretation is consistent with considerable evidence that points to the importance of early sensory experience for the emergence of normal audiovisual integration processes. Neurophysiology studies of cats reared with experimentally manipulated or absent visual input reported abnormal audiovisual multisensory responses in the superior colliculus.[28, 90] Adult humans with a history of transient bilateral visual deprivation in early life show reduced audiovisual multisensory interaction in behavioural studies,[29, 30, 91] and large-scale cross-modal reorganization of the visual cortex as assessed using functional MRI.[92] Interestingly, typically-developing children up to age 7 years have a symmetrically broadened AV simultaneity window similar to that observed in amblyopia, suggesting that the amblyopic AV simultaneity window may represent a persistent juvenile state.[53–56] If the mechanism by which the AV simultaneity window normally narrows through childhood is experience-dependent, then amblyopia may interfere with the calibration and refinement of the cortical processes responsible for AV simultaneity and asynchrony perception. Plausibly, amblyopic visual temporal uncertainty during a critical period of brain development may limit the resolution of AV asynchrony detection, leading to a widened AV simultaneity window.

The view that AV simultaneity perception is altered developmentally by the temporal uncertainty and perceptual latency inherent to amblyopic vision is supported by the lack of a similar effect in monocular adults. Indeed, adults with a history of early enucleation (i.e., removal of one eye) have a normal simultaneity window.[71] This indicates that monocular visual loss alone is not sufficient to alter the simultaneity window, and suggests that impaired but not absent visual input is necessary to disrupt the refinement of temporal audiovisual processes.

While amblyopia is classically regarded as a monocular impairment of spatial vision, the findings of this study, combined with the prior finding of reduced susceptibility to the McGurk effect, indicate an impairment of audiovisual multisensory perception that generalizes beyond speech.[22, 23] In addition to the main finding of a widened AV simultaneity window in amblyopia, subgroup analysis suggested that an accompanying shift in the PSS is dependent on etiology and binocularity. Although the mechanisms are not clear, hypotheses include visual temporal uncertainty and interocular perceptual latency asymmetry. The findings give insight into the developmental calibration of normal multisensory processes, and highlight a previously underappreciated impact of amblyopia beyond vision.

## Supporting information

S1 Dataset Simultaneity judgment data from binocular viewing condition for participants with amblyopia and control participants. (XLSX)

S2 Dataset Simultaneity judgment data from monocular and binocular viewing conditions for subset of participants with amblyopia. (XLSX)
